# Supplementary material for: Structural studies of RFCC tf18 reveal a novel chromatin recruitment role for Dcc1
Source: EMBO Rep. 2017 Feb 10;18(4):558–68. doi: 10.15252/embr.201642825 (PMC5376975; doi:10.15252/embr.201642825)
Supplement: Supplementary file 1 — Appendix [file EMBR-18-558-s001.pdf]

APPENDIX

Contents

Appendix Table S1. X-ray data collection and refinement statistics

Appendix Table S1. X-ray data collection and refinement statistics

**Dataset**	**Ctf18C-Dcc1-Ctf8**	**Dcc1$^{90-380}$ Native**	**Dcc1$^{90-380}$ Peak**	**Dcc1$^{90-380}$ Inflection**
Wavelength (Å)	0.9763	0.92	0.9796	0.9797
Resolution range	60.64 - 2.29 (2.372 - 2.29)	29 - 2.002 (2.074 - 2.002)	79.19 - 2.75 (2.82 - 2.75)	112.75 - 2.96 (3.04 - 2.96)
Space group	$P2_1$	$P2_1$	$P2_12_12_1$	$P2_12_12_1$
Unit cell (Å, °)	58.61 164.22 60.64 90 90.55 90	68.682 100.697 83.495 90 100.67 90	96.69 111.75 112.23 90.00 90.00 90.00	96.82 112.02 112.75 90.00 90.00 90.00
Total reflections	138884 (5577)	288441 (23107)	415363 (28991)	336993 (24158)
Unique reflections	46232 (2926)	71317 (6489)	32295 (2349)	26214 (1898)
Multiplicity	3.0 (1.9)	4.0 (3.6)	12.9 (12.3)	12.9 (12.7)
Completeness (%)	0.90 (0.57)	0.95 (0.87)	99.9 (99.9)	99.9 (99.8)
Mean I/sigma(I)	7.15 (1.31)	8.43 (1.31)	15.7 (3.3)	15.8 (3.8)
Wilson B-factor	43.9	29.31	55.8	45.9
R_{merge}	0.08522 (0.4577)	0.1158 (0.9735)	0.143 (0.822)	0.132 (0.746)
R_{meas}	0.1022 (0.5954)	0.1333 (1.138)	0.149 (0.858)	0.137 (0.778)
$CC_{1/2}$	0.994 (0.732)	0.995 (0.523)	0.996 (0.901)	0.996 (0.881)
No. Sites	-	-	33	-
Figure of merit - acentric (centric)	-	-	0.35491 (0.11335)	
Phasing Power	-	-	1.473	0.391
CC*	0.999 (0.919)	0.999 (0.829)		
Reflections used in refinement	46206 (2922)	71312 (6489)		
R_{work}	0.2018 (0.2967)	0.1903 (0.2844)		
R_{free}	0.2586 (0.3716)	0.2356 (0.3249)		
CC(work)	0.950 (0.820)	0.946 (0.641)		
CC(free)	0.914 (0.591)	0.927 (0.577)		
Number of non-hydrogen atoms	8784	8634		
macromolecules	8518	8112		
Protein residues	1050	1008		
RMS (bonds)	0.01	0.014		
RMS (angles)	1.54	1.41		
Ramachandran favoured (%)	96	97		
Ramachandran allowed (%)	2.8	2.5		
Ramachandran outliers (%)	1.1	0		
Rotamer outliers (%)	2.3	2.8		
Clashscore	9.77	5.43		
Average B-factor	52.99	36.33		
macromolecules	53.07	36.05		
solvent	50.5	40.74		
