# Supplementary material for: Structural studies of RFCC tf18 reveal a novel chromatin recruitment role for Dcc1
Source: EMBO Rep. 2017 Feb 10;18(4):558–68. doi: 10.15252/embr.201642825 (PMC5376975; doi:10.15252/embr.201642825)
Supplement: Supplementary file 2 — Expanded View Figures PDF [file EMBR-18-558-s002.pdf]

Expanded View Figures

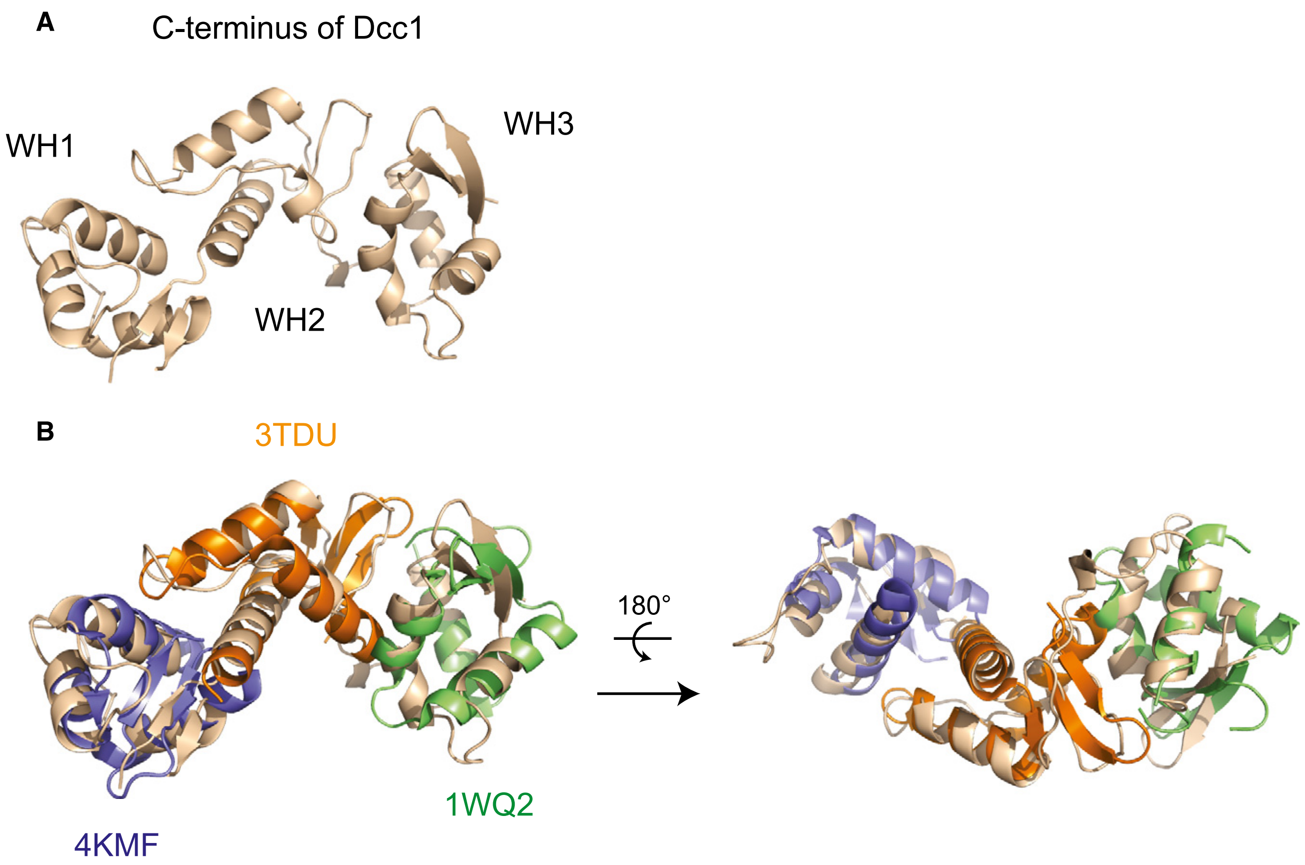

Figure EV1. Structural alignments of the Dcc1 C-terminus with other WH domains.

A The Dcc1 C-terminus is shown in brown and indicated are the WH domains.

B Dcc1 C-terminus is shown in the same orientation as (A) and a structural alignment with three proteins containing WH domains, PKZ (PDB ID: 4KMF, blue), cullin-1 (PDB ID: 3TDU, orange) and DsrD (PDB ID: 1WQ2, green).

© 2017 The Francis Crick Institute

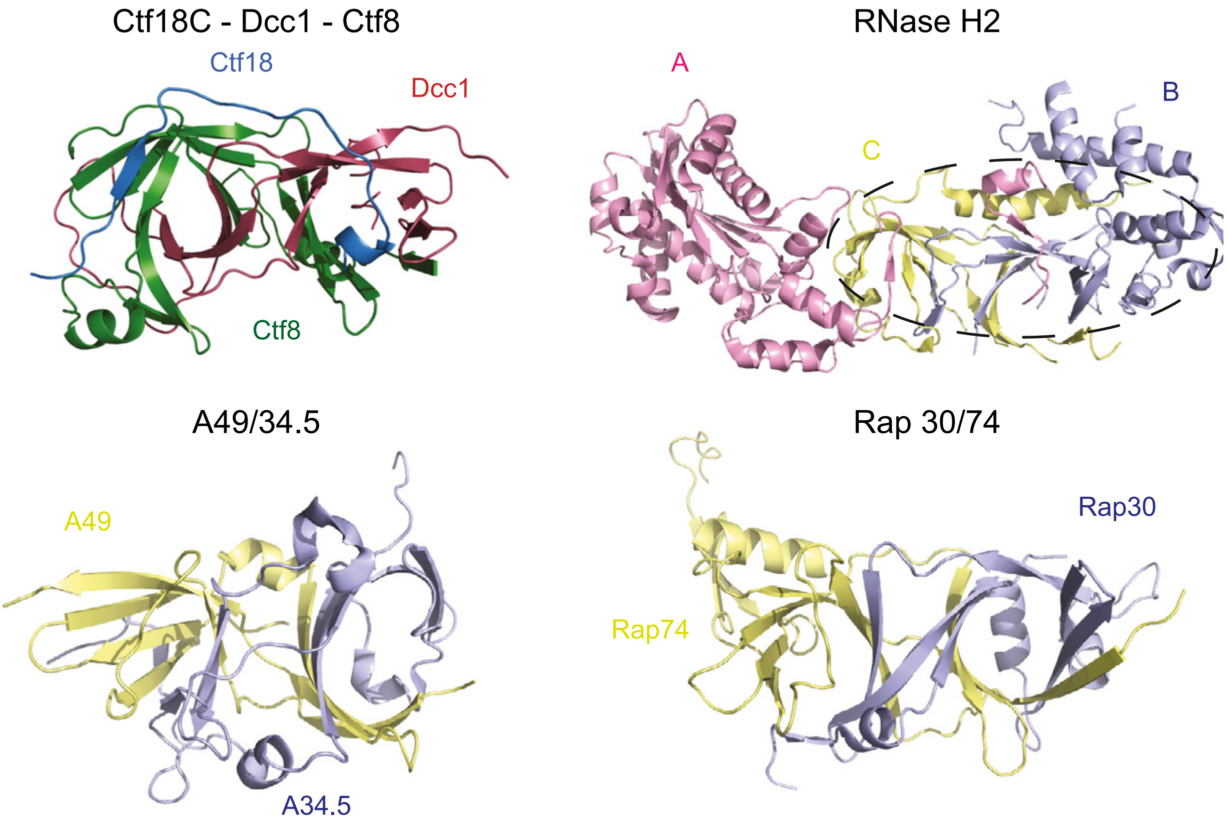

Figure EV2. Similar 'triple β-barrel' dimerisation folds.
RNase H2 complex (3PUF), A49/34.5 (3NFG) and Rap30/74 (1F3U) are presented for comparison with the heterotrimer Ctf18^{C}-Dcc1-Ctf8. The dotted line indicates the area of structural similarity to the Ctf18^{C}-Ctf8-Dcc1 structure.

© 2017 The Francis Crick Institute

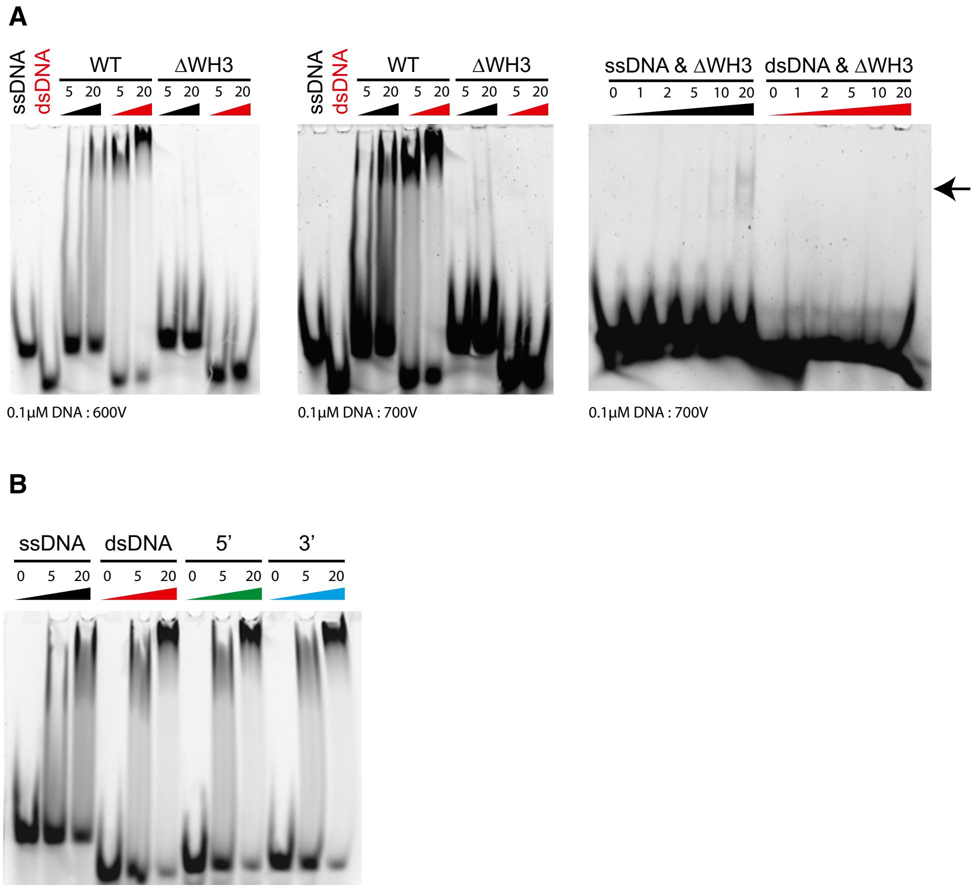

Figure EV3. Additional DNA binding and functional analyses.

A DNA EMSA showing residual binding to ssDNA (indicated by arrow) but not dsDNA by the WH3-deleted Dcc1 construct. Protein concentrations are given in μM.

B DNA EMSA showing binding of Dcc1$^{90–380}$ to 18-bp substrates containing 7-bp 5′ or 3′ overhangs. Protein concentrations are given in μM.

 © 2017 The Francis Crick Institute

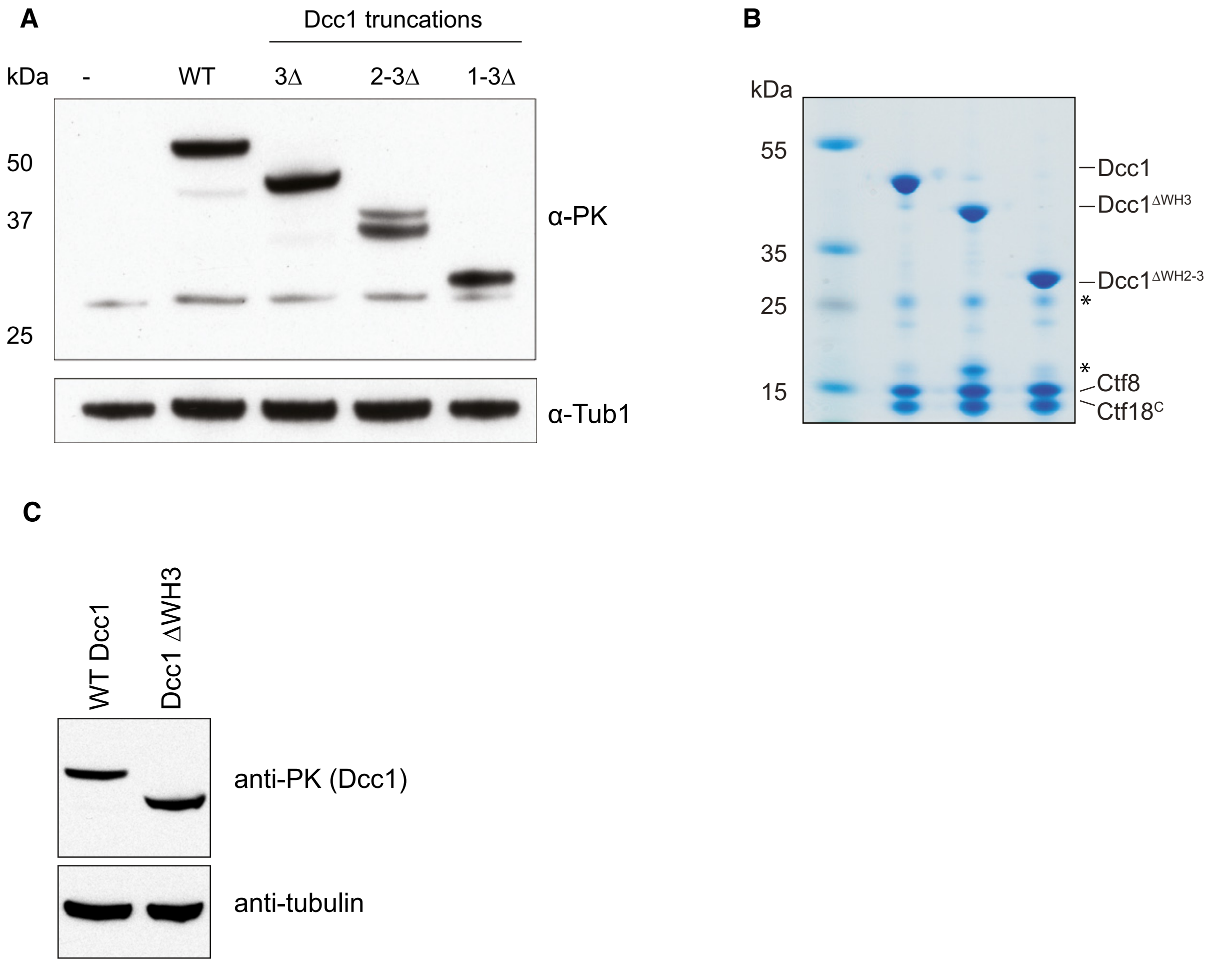

Figure EV4. Expression of and complex formation by Dcc1 WH deletions.

A Western blot showing cellular expression levels of PK-tagged Dcc1 deletions employed for checkpoint activation and sister chromatid cohesion assays. Tubulin is shown as a loading control.

B SDS–PAGE gel of purified recombinant Dcc1-Ctf8-Ctf18^{C} complexes containing indicated Dcc1 deletions. Protein complexes shown were purified in the same way as samples for crystallisation studies. Asterisks indicate impurities in the sample.

C Western blot showing cellular expression levels of Dcc1 deletion employed for ChIP analysis.

© 2017 The Francis Crick Institute
